# Associations Between Asthma and Device‐Based Measured Physical Activity Among Children and Adolescents: The Lolland–Falster Health Study

**DOI:** 10.1111/jpc.70576

**Published:** 2026-09-07

**Authors:** Christian Rønn, Søren Malte Rasmussen, Peter Kamstrup, Therese Lockenwitz Petersen, Jan C. Brønd, Jakob Stokholm, Vibeke Backer, Howraman Meteran, Randi Jepsen

**Affiliations:** ^1^ Lolland‐Falster Health Study, Centre for Health Research Zealand University Hospital, Nykøbing F. Nykøbing F. Denmark; ^2^ Department of Respiratory and Infectious Medicine Copenhagen University Hospital—Bispebjerg Copenhagen Denmark; ^3^ Department of Medicine Zealand University Hospital, Nykøbing F. Nykøbing F. Denmark; ^4^ Steno Diabetes Center Copenhagen Herlev Denmark; ^5^ Department of Clinical Research University of Southern Denmark Odense Denmark; ^6^ Copenhagen Prospective Studies on Asthma in Childhood (COPSAC), Herlev and Gentofte Hospital University of Copenhagen Gentofte Denmark; ^7^ Department of Food Science University of Copenhagen Frederiksberg C Denmark; ^8^ Department of Pediatrics Zealand University Hospital Slagelse Denmark; ^9^ Department of Otorhinolaryngology, Head & Neck Surgery and Audiology Rigshospitalet Copenhagen Denmark; ^10^ Department of Clinical Medicine University of Copenhagen Copenhagen Denmark; ^11^ Department of Respiratory Medicine Copenhagen University Hospital—Hvidovre Hvidovre Denmark; ^12^ Department of Public Health, Environment, Occupation, and Health Aarhus University Aarhus Denmark; ^13^ Department of Respiratory Medicine Zealand University Hospital Roskilde‐Naestved Naestved Denmark

## Abstract

**Aim:**

The aim of this study was to examine whether asthma in children and adolescents is associated with a difference in physical activity (PA) both regarding intensity (sedentary, light, moderate‐vigorous) and behaviour (lying, sitting, standing, walking, running and biking).

**Methods:**

The study sample originated from the Lolland–Falster Health Study, which is a household‐based population study that collected data from February 2016 to February 2020. A sub cohort of the study population was instructed to wear accelerometers. Children and adolescents aged 7–17 with accelerometer data were included in this study. Diagnosis of asthma was self‐reported physician ever diagnosed. A linear mixed model regression analysis was used to evaluate the difference in PA between children and adolescents with asthma compared to children and adolescents without asthma. The analysis was adjusted for age, sex, parental education, tobacco smoking, passive smoking, height, weight and FEV_1_ and within‐family variance was handled as a random effect.

**Results:**

In total, 694 children and adolescents were included in this study, 127 (18%) with a diagnosis of asthma. We did not find a statistical difference in PA between those diagnosed with asthma compared to peers without asthma, neither in PA intensity nor behaviour.

**Conclusion:**

We observed no difference in PA in children and adolescents with asthma compared to peers without asthma.

## 1 Introduction

### 1.1 Background

Asthma is among the most prevalent chronic conditions affecting children and adolescents worldwide. It is characterised by chronic or intermittent airway inflammation, leading to episodes of respiratory symptoms such as wheezing, coughing, chest tightness and shortness of breath, particularly during physical exertion [1].

Despite the potential for exercise‐induced bronchoconstriction, national and international guidelines consistently emphasise the importance of regular physical activity (PA) for children with asthma, citing benefits for both general health and asthma control [1]. A sedentary lifestyle in children with asthma has been linked to poor exercise capacity and obesity [2, 3], as well as poor mental health [4, 5].

In addition to preventing chronic diseases related to a sedentary lifestyle, a meta review concluded that exercise in children and adolescents with asthma is well‐tolerated, increases exercise capacity, improves peak flow as well as exercise‐induced bronchospasm [6]. Additionally, as asthma is largely driven by inflammatory processes, it has been proposed that exercise may improve asthma by reducing inflammation either directly or, alternatively, by weight loss [7]. Thus, exercise could possibly be a nonpharmacological add‐on or alternative to reduce the use of medicine in patients with low medicine adherence.

Questions remain about whether children with asthma are as physically active as children without asthma. The literature presents mixed findings, which vary depending on the method of PA assessment, population characteristics and asthma severity as reported in recent reviews [8, 9, 10].

Studies which employ objective measures such as accelerometers and pedometers suggest that children and adolescents with asthma may engage in similar levels of activity as children and adolescents without asthma, supported recently by data from a British study, encompassing over 6000 seven‐year‐old children, which showed no significant difference in moderate‐to‐vigorous physical activity (MVPA) levels between groups. However, children who had experienced recent hospitalisation for asthma exhibited lower PA levels and were less likely to meet the daily MVPA recommendation of 60 min [11]. A Danish study found similar results in a cohort of five‐year‐old children followed for 4 weeks with an accelerometer. In this cohort, diagnosis of asthma was not associated with reduced PA, but reduced PA was observed in a subgroup of children with increased bronchial responsiveness [12].

There is, however, considerable heterogeneity in study findings: In a German cohort study evaluating adolescents, allergic rhinitis and asthma were associated with lower accelerometer‐measured PA in boys, but not in girls [13]. It should be noted, however, that only a minority of the study population had a diagnosis of asthma. A cross‐sectional study conducted in Brazil which applied both questionnaires and pedometer monitoring demonstrated that asthmatic adolescents walked significantly fewer steps per day, achieved shorter distances on 6‐min walk tests and were more likely to be classified as inactive compared to controls [14]. A similar pattern was observed in a Taiwanese study, where children with moderate to severe asthma reported significantly lower activity levels than their non‐asthmatic peers based on interviews [15].

In summary, only a few studies have used accelerometers in evaluating PA in children and adolescents with asthma, focusing primarily on children. Interestingly, the few small studies that did evaluate PA in adolescents found adolescents with asthma to be less physically active than their healthy peers, contradicting larger studies in children. To our knowledge, no study has evaluated the differences in physical behaviour by accelerometer‐based measurements. A recent meta review stated that the quality of the incorporated studies tempered with the possibility of drawing a clear conclusion and called for future research to incorporate and report more rigorous device‐based PA assessment, with a particular focus on sedentary time [8].

### 1.2 Objectives

The primary objective of this study was to examine whether asthma in children and adolescents is associated with lower levels of MVPA and higher levels of low‐intensity or sedentary PA, compared to peers without asthma. Further, the secondary objective was to assess whether children and adolescents with asthma engage in other PA behaviours, that is, lying, sitting, standing, walking, running, biking, compared to children and adolescents without asthma.

Primary hypothesis: Asthma in children and adolescents is significantly associated with less time spent on MVPA and more time spent sedentary or in light physical activity (LPA), all measured by accelerometer, compared to children and adolescents without asthma.

Secondary hypothesis: Asthma in children and adolescents is significantly associated with a difference in preferred PA behaviour measured by accelerometer compared to children and adolescents without asthma.

## 2 Methods

### 2.1 Study Design

The study sample originated from the Lolland–Falster Health Study (LOFUS) [16]. LOFUS is a household‐based population study that collected data from February 2016 to February 2020. The data collection encompassed age group‐specific questionnaires, physical examinations and collection of biological specimens. The questionnaire for children younger than 11 years was completed by an adult in the household, while participants aged 11 years or older were asked to complete the 11–17‐year questionnaire themselves [17].

As described in detail earlier [18], two Axivity AX3 accelerometers (Axivity, Newcastle, UK) were used to measure PA in LOFUS. Accelerometers were added to the LOFUS data collection in February 2017, initially including households where at least one child (17 years and younger) and one adult consented to wear the accelerometers. From December 2018 onwards, all LOFUS participants were eligible for inclusion. The accelerometers were attached to the skin on the right side of the lower back and on the right thigh, respectively, using adhesive plaster to enable full 24‐h recording. The participants were instructed to wear the accelerometers for 7 consecutive days, including during sleep and water activities and to reapply the accelerometers if they fell off. The raw data from the back were used to identify PA intensities and from the thigh and back to identify PA behaviours, respectively. Estimating subjects' time spent in the commonly used intensity intervals sedentary, LPA and MVPA was based on application of count thresholds that have been previously validated [19]. Estimating the time spent lying, sitting, standing, walking, biking and running for each subject was determined using a simple decision tree in combination with six different signal features generated from the thigh and the back raw acceleration data to identify PA behaviours [19]. The method was original used on adults, but has since been validated with children aged 5–16 years [19, 20]. For the present study, the criterion for a valid day was at least 8 h of wear time during awake time, and only children and adolescents aged 7–17 years of age providing at least 4 valid days were included [19].

### 2.2 Variables

Information about the independent variable was derived from the questionnaires. A positive response to the item ‘physician‐diagnosed asthma/asthmatic bronchitis’ was categorised as ‘Asthma’ and a negative response as ‘Without asthma’. This was an ever diagnosis, not requiring current symptoms.

The dependent variables consisted of different measures of accelerometer‐derived PA intensities and PA behaviours. For the first objective, PA intensities were categorised as time spent sedentary, in LPA, and in MVPA, respectively. The dependent variables for the second objective were time spent on different PA behaviours, that is, lying, sitting, standing, walking, running and biking.

Age and sex were obtained from the Danish Civil Registration System [21]. Height and weight were measured at the physical examination, and body mass index (BMI) was calculated as weight (kg) divided by height (kg/m^2^). Spirometry was also performed at the physical examination, following a standardised procedure that is described in detail elsewhere [22]. Forced expiratory volume in 1 s in litres (FEV_1_ [L]) was used as a measure of lung function. Information about physician‐diagnosed allergic rhinitis, exacerbations of asthma within the last 12 months, and active and passive smoking was obtained from the questionnaires. The educational level of adults was also self‐reported, and in households with more than one adult LOFUS participant, the highest educational level was used as parental education.

### 2.3 Statistical Analyses

Descriptive statistics were performed. Comparison between children and adolescents with and without asthma, respectively, was performed with a chi‐squared test. For continuous data, the normally distributed data were compared with a *t*‐test, and the not normally distributed data were compared with a Mann–Whitney Wilcoxon test.

To control for within‐family variance, a linear mixed model regression analysis was used to evaluate the difference in PA between children and adolescents with asthma compared to children and adolescents without asthma. Within‐family variance was handled as a random effect, and additionally, we adjusted for the following known and suspected confounders: age, sex, parental education, tobacco smoking (children below 11 years of age were considered non‐smokers), passive smoking, height, weight and FEV_1_. Confounders were chosen prior to analysis. FEV_1_ was included as we suspected it independently of asthma status predicts PA. Height and weight were included as separate confounders to better correlate to body composition and lung function. Additionally, a minimal adjusted analysis adjusted for age and sex was performed. Missing data were handled by multiple imputation. Results are reported as difference in time spent daily in minutes with 95% confidence intervals (CIs).

In order to investigate a possible difference between children and adolescents, all analyses were performed both in the complete cohort and stratified by age (< 13 years of age; ≥ 13 years of age).

As sensitivity analyses, a model stratifying for asthma exacerbations within 12 months and a model stratifying for co‐diagnosis of allergic rhinitis were performed.

The statistical analyses were conducted using R 4.5.0 (R Foundation for Statistical Computing, Vienna, Austria).

## 3 Results

### 3.1 Participants and Descriptive Data

A total of 18 949 participants were included in the LOFUS cohort; of these, 4120 participants contributed accelerometer data. Of these, 2304 did not have four full days of accelerometer monitoring, and 994 were not between 7 and 17 years of age, leaving 822 potential participants. Further, 128 did not answer/answered ‘do not know’ on diagnosis of asthma in the baseline questionnaire. Finally, 694 participants were included in this study (see Figure 1). Of the 694 included children and adolescents, 127 (18%) had self‐reported physician‐diagnosed asthma. A slight difference in age, height and FEV_1_ was noted between the groups, with lower values in the asthma group and more with allergic rhinitis; otherwise, no difference in baseline characteristics between participants with and without asthma, respectively, was observed (complete cohort: Table 1, children: Table 2 and adolescents: Table 3).

**FIGURE 1:**
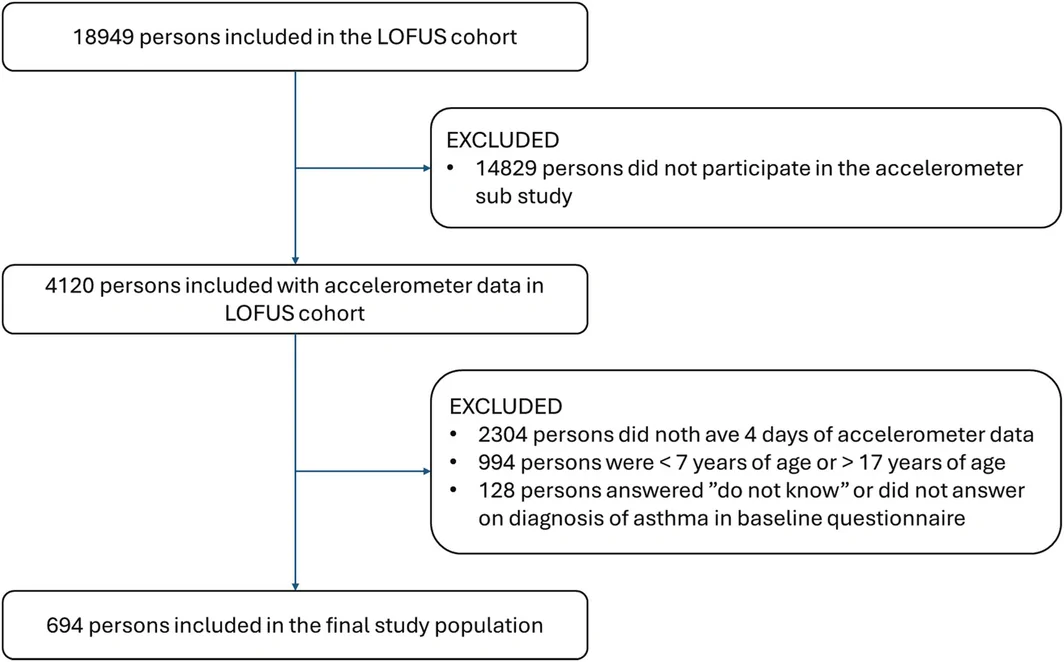
Study flowchart. LOFUS, the Lolland–Falster Health Study.

**TABLE 1:** Baseline characteristics for the complete cohort.

Characteristics	Asthma	Without asthma	*p*
	*n* = 127	*n* = 567	
Age (years), median (IQR)	11.3 (8.5; 13.0)	12.2 (10.0; 14.7)	0.002
Female sex, *n* (%)	66 (52%)	320 (56%)	0.4
FEV_1_ (L), median (IQR)	2.13 (1.65; 2.66)	2.40 (1.89; 3.08)	< 0.001
FEV_1_ of predicted (%), median (IQR)	96 (87; 103)	97 (89; 104)	0.2
Missing	18	77	
Height (cm), median (IQR)	150 (136; 165)	156 (141; 167)	0.006
Weigth (kg), median (IQR)	40.0 (30.1; 54.8)	44.6 (32.1; 57.2)	0.1
BMI (kg/m^2^), median (IQR)	17.9 (15.9; 21.0)	18.2 (16.0; 20.5)	> 0.9
Missing	5	22	
Allergic rhinitis, *n* (%)	36 (28%)	74 (13%)	< 0.001
Missing	< 5	< 5	
Parental education, *n* (%)			0.3
Medium or longer higher education	73 (58%)	303 (55%)	
Short higher or vocational education	47 (37%)	236 (43%)	
Shorter courses or no formal education	5 (4%)	15 (2%)	
Other	< 5	< 5	
Missing	< 5	12	
Active smoking, *n* (%)			0.4
Never	123 (98%)	539 (95%)	
Former	< 5	10 (2%)	
Current	< 5	18 (3%)	
Missing	< 5	0	
Passive smoking, *n* (%)			0.6
Never	85 (67%)	411 (73%)	
Seldom	15 (12%)	54 (10%)	
Often	< 5	10 (2%)	
Daily	24 (19%)	87 (15%)	
Missing	< 5	5	

Abbreviations: FEV_1_, forced expiratory volume in 1 s (L); IQR, interquartile range; *n*, number.

**TABLE 2:** Baseline characteristics for children aged < 13 years of age.

Characteristics	Asthma	Without asthma	*p*
	*n* = 86	*n* = 336	
Age (years), median (IQR)	9.8 (8.0, 11.3)	10.5 (8.9, 11.8)	0.002
Female sex, *n* (%)	45 (52%)	187 (56%)	0.6
FEV_1_ (L), median (IQR)	1.83 (1.59, 2.21)	1.99 (1.66, 2.31)	0.05
FEV_1_ of predicted (%), median (IQR)	97 (87, 103)	95 (88, 101)	0.7
Missing	8	42	
Height (cm), median (IQR)	139 (131, 150)	144 (135, 153)	0.02
Weigth (kg), median (IQR)	34.4 (26.9, 40.6)	34.2 (28.1, 42.9)	0.4
BMI (kg/m^2^), median (IQR)	16.6 (15.6, 19.0)	16.6 (15.1, 18.6)	0.4
Missing	< 5	8	
Allergic rhinitis, *n* (%)	20 (23%)	33 (9.9%)	0.002
Missing	< 5	< 5	
Parental education, *n* (%)			0.4
Medium or longer higher education	49 (58%)	186 (56%)	
Short higher or vocational education	31 (36%)	134 (41%)	
Shorter courses or no formal education	< 5	9 (2.7%)	
Other	< 5	< 5	
Missing	< 5	6	
Passive smoking, *n* (%)			0.2
Never	51 (60%)	239 (71%)	
Seldom	12 (14%)	40 (12%)	
Often	< 5	8 (2.4%)	
Daily	20 (24%)	48 (14%)	
Missing	< 5	< 5	

Abbreviations: FEV_1_, forced expiratory volume in 1 s (L); IQR, interquartile range; *n*, number.

**TABLE 3:** Baseline characteristics for adolescents aged ≥ 13 years of age.

Characteristics	Asthma	Without asthma	*p*
	*n* = 41	*n* = 231	
Age (years), median (IQR)	15.1 (14.1, 16.6)	15.2 (14.1, 16.4)	0.9
Female sex, *n* (%)	21 (51%)	133 (58%)	0.5
FEV_1_ (L), median (IQR)	2.99 (2.56, 3.51)	3.23 (2.86, 3.70)	0.08
FEV_1_ of predicted (%), median (IQR)	92 (83, 103)	99 (90, 107)	0.02
Missing	10	35	
Height (cm), median (IQR)	170 (165, 176)	169 (164, 176)	> 0.9
Weight (kg), median (IQR)	59.8 (54.7, 65.4)	58.9 (52.8, 64.4)	0.5
BMI (kg/m^2^), median (IQR)	20.9 (18.8, 22.2)	20.2 (18.6, 22.3)	0.5
Missing	1	4	
Allergic rhinitis, *n* (%)	16 (39%)	41 (18%)	0.002
Missing	0	1	
Parental education, *n* (%)			0.8
Medium or longer higher education	24 (59%)	117 (52%)	
Short higher or vocational education	16 (39%)	102 (45%)	
Shorter courses or no formal education	< 5	6 (2.7%)	
Other	< 5	< 5	
Missing	< 5	12	
Active smoking, *n* (%)			0.5
Never	38 (93%)	203 (88%)	
Former	< 5	10 (4.3%)	
Current	< 5	18 (7.8%)	
Passive smoking, *n* (%)			0.6
Never	34 (83%)	172 (76%)	
Seldom	< 5	14 (6.2%)	
Often	< 5	< 5	
Daily	< 5	39 (17%)	
Missing	< 5	< 5	

Abbreviations: FEV_1_, forced expiratory volume in 1 s (L); IQR, interquartile range; *n*, number.

### 3.2 Primary Results

For the complete cohort considering both children and adolescents no difference in PA intensities was observed between children and adolescents with asthma and children and adolescents without asthma for either sedentary PA: difference in favour of participants with asthma 20.8 min/day (95% CI: −5.6; 47.2), LPA: difference −2.6 min/day (95% CI: −10.6; 5.4) or MVPA: difference 15.8 min/day (95% CI: −23.4; 54.9) in the full adjusted analysis, for the minimal adjusted analysis see Table 4.

**TABLE 4:** Results for the complete cohort.

Activity	Adjusted analysis				Minimal adjusted analysis			
	Difference	95% CI		*p*	Difference	95% CI		*p*
Sedentary	20.8	−5.6	47.2	0.1	19.0	−7.1	45.2	0.2
LPA	−2.6	−10.6	5.4	0.5	−2.9	−11.1	5.2	0.5
MVPA	15.8	−23.4	54.9	0.4	17.6	−21.2	56.3	0.4
Lying	13.7	−42.3	69.6	0.6	10.9	−44.5	66.3	0.7
Sitting	1.0	−29.1	31.1	0.9	−3.2	−33.2	26.9	0.8
Standing	−9.7	−27.3	7.8	0.3	−10.4	−28.0	7.1	0.2
Walking	−0.3	−7.8	7.2	0.9	−0.6	−10.3	9.2	0.9
Running	5.7	−0.9	12.3	0.09	6.4	−0.1	13.0	0.06
Biking	4.2	−1.0	9.5	0.1	4.8	−0.4	10.0	0.08

*Note:* Difference in time (min/day) spent on different activities between children and adolescents with and without asthma. Difference in favour of children and adolescents with asthma.

Abbreviations: CI, confidence interval; LPA, light physical activity; MVPA, moderate‐vigorous physical activity.

In the subgroup considering children aged < 13 years of age and the subgroup considering adolescents aged ≥ 13 years of age, the results mimicked those of the complete cohort, that is, no difference in PA intensities was observed, for results for the children see Table 5 and for the adolescents see Table 6.

**TABLE 5:** Results for subgroup of children aged < 13 years of age.

Activity	Adjusted analysis				Minimal adjusted analysis			
	Difference	95% CI		*p*	Difference	95% CI		*p*
Sedentary	13.5	−17.7	35.2	0.5	8.3	−17.6	34.2	0.5
LPA	48.0	−97.3	90.8	0.5	−2.3	−11.6	6.9	0.6
MVPA	22.4	−28.0	59.8	0.5	13.1	−30.2	56.4	0.6
Lying	33.1	−57.4	72.2	0.8	2.8	−61.4	67.0	0.9
Sitting	18.3	−35.9	35.9	1.0	−2.2	−37.8	33.4	0.9
Standing	11.0	−36.8	6.3	0.2	−13.7	−35.1	7.7	0.2
Walking	5.2	−10.7	9.6	0.9	0.4	−9.7	10.4	0.9
Running	3.7	−6.0	8.5	0.7	1.9	−5.3	9.1	0.6
Biking	2.9	−5.8	5.4	0.9	0.1	−5.4	5.7	1.0

*Note:* Difference in time (min/day) spent on different activities between children with and without asthma. Difference in favour of children with asthma.

Abbreviations: CI, confidence interval; LPA, light physical activity; MVPA, moderate‐vigorous physical activity.

**TABLE 6:** Results for subgroup of adolescents aged ≥ 13 years of age.

Activity	Adjusted analysis				Minimal adjusted analysis			
	Difference	95% CI		*p*	Difference	95% CI		*p*
Sedentary	29.0	−9.4	104.4	0.1	36.3	−19.7	92.3	0.2
LPA	7.7	−18.8	11.4	0.6	−4.8	−20.1	10.4	0.5
MVPA	40.0	−56.9	100.0	0.6	21.9	−54.0	97.9	0.6
Lying	55.8	−90.3	128.6	0.7	16.6	−88.9	122.1	0.8
Sitting	28.9	−56.1	57.3	1.0	−8.1	−63.4	47.3	0.8
Standing	15.6	−38.8	22.2	0.6	−5.2	−35.3	24.9	0.7
Walking	5.8	−16.9	5.8	0.4	−4.6	−15.8	6.6	0.4
Running	7.3	1.4	30.0	0.05	17.0	3.0	31.0	0.03
Biking	5.9	2.9	26.0	0.03	14.6	3.2	26.1	0.02

*Note:* Difference in time (min/day) spent on different activities between adolescents with and without asthma. Difference in favour adolescents with asthma.

Abbreviations: CI, confidence interval; LPA, light physical activity; MVPA, moderate‐vigorous physical activity.

### 3.3 Secondary Results

The investigated potential differences in time spent lying, sitting, standing, walking, running and cycling, respectively, revealed no difference between the groups, as reported in Table 2.

### 3.4 Sensitivity Analyses

As a sensitivity analysis, a subgroup analysis of participants with exacerbation of asthma was performed; however, only 19 participants had experienced an exacerbation within the last 12 months, and the analysis found no association with either PA intensity or behaviour (data not shown). Also, stratifying for co‐diagnosis of allergic rhinitis did not change the results (data not shown). Finally, in the sensitivity analysis using FEV_1_ of predicted as a marker of asthma, no association with either PA intensity or behaviour was found (data not shown).

## 4 Discussion

### 4.1 Key Results

This study aimed to compare PA behaviour and intensities between children and adolescents with and without asthma. Our results showed no significant difference between the two groups, neither based on activity intensity nor on specific behaviours. However, in a subgroup analysis considering adolescents aged ≥ 13 years, a diagnosis of asthma was significantly associated with increased time spent running and cycling compared to adolescents without asthma.

### 4.2 Interpretation

Some recent studies have suggested that children and adolescents with asthma are less active than peers without asthma, especially in participants with symptomatic asthma and in adolescents, respectively, while no association between participants with well‐controlled asthma and reduced PA seems to be present [8, 9, 10, 11]. Our study, offering a large, accelerometer‐measured cohort of children and adolescents, generally agreed with these recent studies where no association between well‐controlled asthma and reduced PA was found. However, in a subgroup of adolescents aged ≥ 13 years, asthma was significantly associated with increased time spent running and cycling compared to adolescents without asthma.

A German cohort study and a Brazilian cross‐sectional study both utilising device‐measured assessment of PA found that adolescents with asthma were less active compared to adolescents without asthma [13]. We found no association between PA intensities and asthma in either the complete cohort of children and adolescents or the subgroup consisting of adolescents aged ≥ 13 years. However, in the adolescent subgroup we noted that both time spent running and time spent cycling were higher in adolescents with asthma than in those without asthma, conflicting with the cited studies. It should be noted that these results might be chance findings, as the results were derived from an exploratory subgroup analysis and that multiple testing is not accounted for. However, one explanation might be that those with a more active lifestyle might be more likely to get diagnosed with asthma than those with a more sedentary lifestyle.

Several studies have indicated that children with asthma are more likely to avoid physical activity due to concerns about exacerbation of asthma symptoms, such as shortness of breath, wheezing or coughing during exertion and challenges with asthma management [23]. These findings suggest that asthma might influence the type or intensity of PA but not necessarily the overall activity levels. However, our findings suggest no difference in either intensity or type of PA. Other studies have found similar results.

A recent review found no significant difference in PA between children with well‐controlled asthma and healthy controls. The review included studies both with accelerometer‐assessed PA, similar to our study, and studies with self‐reported data on PA [8].

A large British cross‐sectional study also observed no significant difference in accelerometer‐measured PA between children with asthma and those without, particularly in environments where asthma management was optimised [11]. Likewise, in our study, with only 19 of 127 children and adolescents reporting exacerbations of asthma 1 year prior to study entry, which likely contributed to the absence of a difference in overall PA between the two groups. This supports the notion that advancements in asthma treatment and management can help children with asthma maintain comparable activity levels to their peers.

Our study adds to the limited data on accelerometer‐measured PA in children and, especially, adolescents focusing on time spent sedentary, LPA and MVPA as called for by a recent meta‐analysis [8]. Also, we examined time spent on different PA behaviours with no sign of reduced time spent lying, sitting, standing, walking, running and biking. In the current study cohort, only 19/127 participants with asthma reported an exacerbation of asthma 12 months prior to study entry. We hypothesise that asthma in children and adolescents with a low prevalence of exacerbations does not influence PA and encourage future studies to include children and adolescents with more symptomatic asthma.

### 4.3 Strengths and Limitations

An important strength is the use of accelerometer data to measure PA, providing a more accurate assessment of PA than self‐reported data. Accelerometers are useful tools for capturing both the intensity and duration of PA, and they offer an objective means of comparing activity levels and PA behaviours between groups. Also, important confounders were measured at participation in the study, including spirometry data, height and weight. Furthermore, we had access to self‐reported data on tobacco use and exposure and parental educational level.

Our study has some limitations that should be addressed. First, data on asthma and exacerbations of asthma were self‐reported and therefore might not be precise. Also, we cannot determine whether the diagnosis was still ongoing at the time of visit. Most children experiencing early life asthmatic episodes will outgrow their symptoms before school age, and only a few participants reported current respiratory symptoms, making it difficult to determine whether the small number of children and adolescents with poorly controlled asthma displayed reduced PA due to large statistical uncertainty. Second, we did not have access to medical records or prescription databases and no self‐reported data on daily respiratory symptoms, making it difficult to differentiate disease severity. We did, however, have access to self‐reported exacerbations 12 months prior to study entry which 19/127 children and adolescents with asthma reported. Third, participants were not systematically evaluated for asthma; therefore, participants with a more inactive daily life, perhaps because of asthma, may to a larger extent be undiagnosed in comparison to participants with a more active daily life experiencing respiratory symptoms leading to physician evaluation. Also, adults participating in the LOFUS cohort have been reported to have better health status than non‐participating peers. The same may be true for participating children and adolescents [24].

## 5 Conclusion

In conclusion, our study found no significant difference in accelerometer‐measured PA intensity or behaviour between children and adolescents with asthma compared to peers without asthma.

## Funding

The study was financed by grants from Region Zealand Health Scientific Research Foundation (R37‐A1569). The funding sources had no influence on the study design, data collection, analysis or reporting of data.

## Ethics Statement

Region Zealand's Ethical Committee on Health Research (SJ‐421) approved the Lolland–Falster Health Study. LOFUS and the present study are registered in the Danish Data Protection Agency (p‐2024‐16360 and p‐2024‐16538). LOFUS is registered in Clinical Trials (NCT02482896). Written informed consent was obtained at the physical examination. Parents or other holders of custody signed the consent form for participants aged 0–14 years [16].

## Conflicts of Interest

The authors declare no conflicts of interest.

## Data Availability

The data that support the findings of this study are available from LOFUS, Statistics Denmark and Danish Health Data Authorities. Restrictions apply to the availability of these data, which were used under license for this study. Data are available from the author(s) with the permission of LOFUS, Statistics Denmark and Danish Health Data Authorities.
